# The National Medical Union (Official)

**Published:** 1914-05-23

**Authors:** M. Macnaughton Jones


### THE NATIONAL MEDICAL UNION (Official).

Offices: 346 Strand. Tel.: City 8444.

### MEDICAL FORETHOUGHT IN STATE MEDICINE.

Abstract of Paper by Dr. M. MACNAUGHTON JONES, Junr. (*read before the Hampstead Non-Panel Association, April* 30, 1914).

I must first thank you for the very great honour your committee conferred on me when it invited me to open at your first general meeting a discussion on the effects of the Insurance Act upon the ethics of the profession. The more I read the deeper becomes my conviction that for a long while the ship has been totally unseaworthy; that it has had neither pilot nor captain; that no one studied the social currents which were bearing it along; that while it was drifting the crew were quarrelling; that to deal with the past now is to let it detain us, and that our duty is to deal with the stern realities of to-day. We should furnish facts and suggestions to statesmen lest we find ourselves again crying over spilt milk, satiating the public with profitless discussion over ills which we cannot redress. Therefore at the last minute I asked your secretary to let me substitute the present subject for that already chosen.

It is undesirable, as well as impossible, to discuss detail in a short paper, since discussion tends to circle round unimportant details, instead of concentrating on broad principles. There are three lines along which it has been suggested reform should run: remuneration for services rendered; a modification of the present system; a part or whole-time service. The first, though otherwise an ideal system, is so open to abuse that I think we may eliminate the possibility of its adoption. Of the other two, which is more likely to draw able and ambitious men into the profession, make work, originality and ability the qualifications for success, and induce the rank and file, as well as the leaders of the profession, to give of their best to the State?

Dissatisfaction is often expressed at the reputed size of many incomes, the ease with which they are made, and the disparity between them and those of smaller men. But it involves time, risk, and exceptional effort to reach the fruit at the top of the tree, and more blanks than prizes await those who essay to obtain it. It is this fruit, moreover, which tempts men of ability and ambition of all classes into the profession, and helps to secure its social position. Under our present system, when a vacancy occurs on a hospital staff, the ablest candidate is selected by men, each of whom has been himself selected for his ability. But to place him on the staff is not sufficient. On his contributions to literature, his skill as a teacher, an operator, or a physician, or on his work in the laboratory his success depends. Nor is that all; the work of to-day is superseded, and, unless really remarkable, is forgotten to-morrow. The tribunal before which he stands, whether a body of senior students or general practitioners, is nearly always a competent and fair one. Every operation or diagnosis is criticised by those on whose support he depends. Everything professional which he does or says finds its place in the balance for or against him. A distinguished career as a student and examinational honours qualify him to take his place among consultants, but for success he must rely upon continuous effort and accomplishment. Compare the position of such a jury to give a verdict, the motives which actuate them, and the direct appropriateness of the award, with promotion by an officer or board, on brief, and possibly biassed, reports, even if merit were the sole consideration. We know, however, that the only way yet devised to check favouritism and influence in a public service is to promote, as far as possible, by seniority. Where administrative work is required a public service may be all that is desired, but it seldom calls forth, as does never-ending competition, supreme and continuous effort to excel.

I have dealt so far with the effect of a whole-time State service upon the progress of medicine and its leading representatives. I now turn to compare it with subsidised private general practice. One disadvantage in a State system would be the removal of a practitioner from his patients as he obtained promotion. In the prolonged discussion which has taken place the only common ground between the exponents and opponents of a State system appears to be that in a private practice it is to the doctor's interest to please patients, whereas, if anything, the opposite is the case in a State system.

The advocates of State medicine seem to interpret pleasing the patient as fawning, flattering, and pandering to hysteria and malingering. The majority of men fear illness, not because of the pain or distress of which it is the direct cause, but on account of the other serious consequences it may entail, both to themselves and to all dependent on them. Frequently there is nothing which so retards recovery or is so injurious as mental anxiety. The nervous patient in whom the doctor has inspired confidence, and who leaves his consulting room satisfied that no serious trouble could have escaped his attention, is already on the road to recovery, and the doctor has secured the most valuable of all conditions—namely, the faithful fulfilment of his instructions.

Sir John Collie, in his lecture on State Medical Service, referring to free choice of doctor, said: "It means that a doctor is selected for his power of ingratiating himself, or on account of his having a thing I am told I never had, a good bedside manner—(laughter)—rather than for the capacity of treating disease. A premium is placed upon advertising, and the doctor with the most pleasing manner, and greatest readiness to give certificates—(loud laughter)—is most successful." Does Sir John Collie mean to suggest that he is exceptional because he does not gently stroke the hand of a labourer or working woman, when called in to attend them; or that he is singular because, when he is ushered into the august presence of some poor charwoman, he does not copy the grotesque mannerisms sometimes indulged in on the stage by actors when playing the part of physician to a queen or duchess? Or does he mean that the women and children of the insured classes should know neither nervousness nor diffidence, and that the physician who cultivates courtesy and tact in interrogation and examination of them is unworthy of the profession?

Whether their complaints are imaginary or real, in my experience, the surest way of pleasing patients is by inspiring confidence and restoring health. The practitioner who succeeds in doing so will gain more patients in a month than he will lose in a year by refusing to countenance malingering. In private practice, in the presence of competition, as it exists, each success or failure counts; nothing succeeds like success in diagnosis and treatment; practitioners know it, and could have no stronger stimulus to effort.

Anyone starting in a poor neighbourhood, unless he suffers from some exceptional personal disability, who gives adequate time and thought to all his patients, will have as much as he can do; but his income will be such that no competent man will spend the time necessary to qualify in order to earn it. No doubt many men do give panel patients adequate attention, but their panel lists must necessarily be limited, and the majority of the insured have to choose men who are willing to do the best they can in the circumstances.

Our object should be to secure competition under fair and reasonable, instead of under the present impossible, conditions, so as to bring out all that is best in the individual.

A serious handicap which men in poor-class practices cannot escape is the difficulty of employing the instruments or appliances frequently required to make an accurate diagnosis. It is impossible, unaided, to sterilise hands and instruments in less than fifteen minutes. I am considerably understating the time given in text-books. A degree of surgical cleanliness which involves very little trouble in a good-class practice, and none in a hospital where hot and cold sterile water, lotions, utensils, sterile dressings, and towels are at hand, becomes impossible in a very poor-class practice. Here the practitioner has not only to improvise, but to prepare everything in advance, and once his hands are prepared, may be embarrassed by the most trivial oversight in preparation. Leaving out of consideration the expense incurred in the provision and upkeep of specula and other appliances, the time involved in cleaning and sterilising them renders their employment either unsafe or impracticable.

Allowing for these unavoidable disabilities, contrast a visit to a consultant with a visit to the practitioner in some panel surgeries. The consultant insists on obtaining, possibly briefly, a clear and accurate conception of the history of the case. The striking feature of the visit is the thoroughness of his examination. He keeps a record, sufficient if short. Finally he gives advice with or without a prescription. There are cases in which the condition is obvious to the expert at a glance, but even then the causes are if possible ascertained, and he takes care that the details of treatment are clearly understood. There are surgeries, on the other hand, in which men see a constant stream of patients, giving none of them the necessary examination, giving little consideration to or variation in treatment, keeping practically no records, and unable to grasp sufficient details of the case to form any opinion, from personal observation, either of its progress or of the effect of the treatment employed. The consultant who carefully ascertains or observes the first symptoms which manifest themselves, is acquiring skill in early diagnosis. But the man whose diagnosis is a happy or unhappy inspiration, and whose treatment is a routine prescription, is acquiring skill in neither diagnosis nor treatment.

Furthermore, no one reads more intently than the general practitioner who with a full sense of the consequences of error is endeavouring to master the diagnosis and treatment of a case; but if he has no leisure for reading, instead of spending his life acquiring knowledge, he is gradually losing his grasp on methods which have probably been superseded, but with the improvements on which he is not acquainted. The majority of serious diseases to be successfully treated must be recognised at a stage at which they may be easily confounded with trivial ailments, when the diagnosis is difficult and requires both skill and time.

A great deal of medical attendance on the poor has for many years been a reproach to medicine and a blot upon civilisation. This has been recognised and resented by all classes of the medical profession. It was the best that could be done in the conditions, but in many instances it was all but valueless. The Chancellor raised hopes that in these poorer practices he would enable us to do justice to ourselves, to our patients, and to the State; and it was the recognition that the new conditions, while possibly better than the old, would not achieve this end that caused so much dissatisfaction.

Prior to the Act insured classes paid from threepence to 3s. per visit. All now pay from ninepence to 1s., and though many ulterior considerations, such as attendance on relatives, complicate the issue, broadly speaking those who formerly paid threepence get better treatment, while those who paid 3s. do not get as good. Had the Chancellor accepted a lower income limit, incomparably greater benefit would have resulted, at a much less cost to the State. His difficulties were real, but might have been overcome.

Sir John Collie accepted Sir William Plender's conclusions that the medical profession received 4s. 2d. per person per annum, and said that "under these circumstances I cannot help thinking that when so generous a sum as 7s. for medical attendance alone was decided upon, the Commissioners should at least have decided upon a complete service." Out of this generous sum he thinks that laboratories, bacteriological investigations, surgical treatment, and special treatment for eyes and ears ought also to be provided. The fundamental fallacy, that genuine medical attendance was obtainable for the fees formerly paid by club patients, appears to me to be the cause of all our trouble, and until we dispel that fallacy progress is impossible.

Every student wonders at the mistakes made by general practitioners whose patients drift into the hospital at which he happens to be. When he enters practice such cases continue to obtrude themselves on his notice. He then sees that he is no abler than the men whose mistakes are so obvious, but that in a certain class of case they make no serious attempt at diagnosis. Necessarily an undue proportion of the patients who drift to hospital, having failed to obtain relief from private treatment, are the victims of such men; and students, nurses, patients, and the public in general get a very unfair impression of the ability of the general practitioner.

Committees of independent medical men should insure adequate attention being given to nearly every case. They might deal gently with incapacity, but should not be blind to culpable shortcomings. Effective medical control of medical service is absolutely indispensable to the success of any scheme, and medical control is indispensable to the profession if control is to be a reality. Only medical men can differentiate between mistakes, which are the daily lot of consultants and practitioners alike, and culpable carelessness. The presence of laity on the medical committees would hamper discussion, though the insured will always claim and should have the right of appeal to a body the majority of whom are laymen. No statesman will place the control in medical hands merely to satisfy the prejudices or traditions of our profession, but he will do so if convinced it is the surest way of exposing culpable neglect, as well as the most just method of dealing with medical men.

**(Continued on p. 3 of the "Institutional Worker."**

